# Phase Angle Is a Potential Novel Early Marker for Sarcopenia and Cognitive Impairment in the General Population

**DOI:** 10.1002/jcsm.13820

**Published:** 2025-05-08

**Authors:** Kentaro Ikeue, Hisashi Kato, Masashi Tanaka, Hajime Yamakage, Sayaka Kato, Masayo Iwasa, Kan Oishi, Yuiko Yamamoto, Megumi Kanasaki, Izuru Masuda, Kojiro Ishii, Noriko Satoh‐Asahara

**Affiliations:** ^1^ Department of Endocrinology, Metabolism, and Hypertension Research Clinical Research Institute, NHO Kyoto Medical Center Kyoto Japan; ^2^ Graduate School of Health and Sports Science Doshisha University Kyoto Japan; ^3^ Department of Rehabilitation Health Science University Yamanashi Japan; ^4^ Faculty of Health and Sports Science Doshisha University Kyoto Japan; ^5^ Takeda Hospital Medical Examination Center Kyoto Japan; ^6^ Diabetes Medicine, Mitsubishi Kyoto Hospital Kyoto Japan

**Keywords:** bioimpedance analysis, cognitive decline, memory, muscle quality, phase angle

## Abstract

**Background:**

Sarcopenia is associated with an increased risk for dementia. This study aimed to elucidate the relationship between sarcopenia‐related indices and cognitive decline in the general population.

**Methods:**

This was a cross‐sectional study involving 263 participants (163 men with a median age of 60 years [interquartile range = 53–70] and 100 women with a median age of 58 years [interquartile range = 49–68]) who underwent a general health examination. Sarcopenia‐related indices included appendicular skeletal muscle mass (ASM)/height^2^, ASM/body mass index, handgrip strength (HGS), HGS/upper extremity skeletal muscle mass and phase angle (PhA). We examined the associations between these indices and cognitive function using the Japanese version of the Montreal Cognitive Assessment (MoCA‐J).

**Results:**

Higher PhA, an indicator of muscle quality, was associated with a lower risk of mild cognitive impairment (MCI) in women (adjusted odds ratio = 0.28 [95% confidence interval, 0.10–0.78], *p* = 0.014), whereas the other sarcopenia‐related indices showed no significant association with MCI in both sexes. The PhA of women was positively associated with the MoCA‐J scores (*β* = 0.27, *p* = 0.005). Moreover, the PhA of women showed a positive correlation with cognitive subdomains, including memory (*r* = 0.22, *p* = 0.031), which is one of the earliest manifestations of cognitive impairment. The PhA in men was also positively correlated with memory (*r* = 0.24, *p* = 0.002).

**Conclusions:**

PhA is a potentially novel index for detecting the risk of sarcopenia and cognitive decline in the general population.

## 1 Introduction

Sarcopenia is a condition characterized by the progressive loss of skeletal muscle mass and function [1, 2]. Sarcopenia is one of the most prevalent health issues in older adults and increases the risk of adverse health outcomes, such as frailty, falls and fractures, hospitalization and mortality [1, 2]. Accumulating evidence has further shown a detrimental relationship between sarcopenia and dementia, which is also a serious global health issue [2]. However, there are currently no effective indices for the early detection of individuals at risk of both sarcopenia and cognitive dysfunction.

Thus far, several international research groups have reported diagnostic criteria for sarcopenia, but the diagnostic technique is yet to be standardized due to its diverse and controversial aspects [1]. Although the cut‐off values of sarcopenia‐related indices also differ among research groups, the common indices in the diagnostic criteria include muscle mass, muscle strength, and physical performance [1]. Accordingly, it is recommended to measure these three parameters for the diagnosis of sarcopenia, whereas the appropriate selection of diagnostic criteria would depend on the race and the feasibility of the measurements [1].

In addition to these indices, growing evidence has highlighted the novel significance of the phase angle (PhA) in the diagnosis of sarcopenia [1, 3, 4, 5]. PhA is an indicator of cellular health that reflects intracellular and extracellular fluid status, cellular nutritional status, cell membrane integrity and cell function [6]. PhA and handgrip strength (HGS) were reported to be associated with malnutrition, a risk factor for sarcopenia [1], but PhA proved to be a more sensitive indicator than HGS [7]. Other studies have shown that PhA is lower in individuals with sarcopenia than in those without sarcopenia [3, 5]. In a previous study, we revealed that PhA is an index of muscle quality and is useful for detecting sarcopenia [5]. These findings suggest that muscle quality, as well as muscle mass, strength and physical performance, would be valuable indices for the diagnosis of sarcopenia.

Recent extensive studies have further demonstrated the relationship between skeletal muscle and cognitive function [2, 8]. Exacerbation of sarcopenia‐related indices, such as muscle mass, strength, and physical performance, is associated with cognitive impairment [2, 8, 9, 10]. Muscle quality assessed using PhA has also been reported to decrease with cognitive decline in patients with mild cognitive impairment (MCI) and Alzheimer's disease (ad) [11, 12]. Furthermore, the risk of cognitive impairment was reported to be elevated in older adults with sarcopenia than in those without sarcopenia [13]. The prevalence of sarcopenia is higher in individuals on the ad continuum (preclinical ad, MCI due to ad and ad dementia) than in those without ad symptoms, and sarcopenia is associated with faster progression of AD [14]. Therefore, sarcopenia‐related indices, including muscle quality, could be implicated in cognitive decline.

To explore novel indices that allow the early identification of individuals at risk of both sarcopenia and cognitive impairment, it is more desirable to investigate those markers in the general population than in individuals already diagnosed with sarcopenia or dementia. Furthermore, since dementia influences multiple subdomains of cognitive function (e.g., memory, language and executive function) [2], identifying which of the sarcopenia‐related indices are related to which of the cognitive subdomains could elucidate the dementia continuum and aid the development of appropriate interventions. In the present study, we investigated the possibility of using sarcopenia‐related indices, including muscle quality, as novel markers of cognitive decline in the general population.

## 2 Methods

### 2.1 Study Participants and Design

This cross‐sectional study included 267 individuals aged ≥40 years who underwent a comprehensive health examination at Takeda Hospital Medical Examination Center in Japan between August and November 2022. Participants received oral and written information about this study, and informed consent was obtained from the participants prior to the study. We did not recruit individuals with pacemakers, limb defects or pregnancies (including women with suspected pregnancies). All participants completed a questionnaire and were interviewed by a public health nurse regarding their medical history, family history, oral medications, and lifestyle information (eating habits, smoking status, alcohol consumption and exercise habits).

This study was approved by the Ethics Committee for Human Research at the National Hospital Organization Kyoto Medical Center (Approval No. 21‐082) and Takeda Hospital Group (approval no. 2125). This study was conducted in accordance with the principles of the Declaration of Helsinki and the ethical guidelines for medical and health research involving human participants. This study was registered in the University Hospital Medical Information Network (UMIN) system (UMIN Study ID: UMIN000048056). All data were encrypted, anonymized and stored under password protection in the device.

### 2.2 Survey and Laboratory Tests

Age, sex and smoking status (current smoker or non‐smoker) were obtained from each participant using a self‐administered questionnaire. Height and body weight were measured in increments of 0.1 cm and 0.1 kg, respectively. Body mass index (BMI) was calculated as body weight (kg) divided by the square of the height (m^2^).

Systolic and diastolic blood pressures (SBP and DBP, respectively) were measured using a sphygmomanometer after 5 min of rest. The mean of two blood pressure measurements was used for the analyses. Venous blood samples were collected in the morning after an overnight fast to measure fasting plasma glucose (FPG), haemoglobin A1c (HbA1c), triglyceride (TG), high‐density lipoprotein cholesterol (HDL‐C) and low‐density lipoprotein cholesterol (LDL‐C) levels. Hypertension, diabetes and dyslipidemia were diagnosed according to the criteria of each academic society: Hypertension was defined as SBP ≥ 140 mmHg and/or DBP ≥ 90 mmHg or taking medications for hypertension [15]; diabetes was defined as FPG ≥ 126 mg/dL and/or HbA1c ≥ 6.5% or taking medications for diabetes [16]; and dyslipidemia was defined as TG ≥ 150 mg/dL and/or HDL‐C < 40 mg/dL and/or LDL‐C ≥ 140 mg/dL or taking medications for dyslipidemia [17].

### 2.3 Body Composition, Muscle Strength Tests and Calculation of Sarcopenia‐Related Indices

Whole‐body and segmental composition data such as appendicular skeletal muscle mass (ASM) and PhA were obtained by measuring bioelectrical impedance using a multifrequency segmental body analyser (MC‐780A‐N, TANITA, Tokyo, Japan). The system uses currents at three different frequencies (5, 50 and 250 kHz) to achieve high accuracy. Resistance and reactance values of 50 kHz were adopted for the lower extremities, and PhA was calculated using the following equation [18]:

PhA (deg) = −arctangent (reactance/resistance)*(180/*π*)

In this bioelectrical impedance analysis (BIA), individual data (age, sex, and height) were input into the device, followed by measuring the bioelectrical impedance of the participant with an overnight‐fasting state while standing on foot‐electrodes and holding hand‐electrodes [4]. All measurements were performed by well‐trained personnel following the system guide.

Skeletal muscle mass index (SMI) and ASM/BMI were calculated using ASM (kg) divided by the square of height (m^2^) or BMI (kg/m^2^), respectively.

HGS was measured twice for each hand using the Smedley grip force system (Grip‐D, Takei Equipment Company, Tokyo, Japan) in a standing position, and the maximum value was included in the analyses. The HGS per unit of upper limb skeletal muscle mass (USM) was calculated by dividing the HGS (kg) by the USM (kg).

The cut‐off values of sarcopenia‐related indices remain a matter of debate [1], and this study aimed to explore novel markers identifying individuals at risk of sarcopenia and cognitive decline in the general population rather than in patients with sarcopenia and/or dementia. Therefore, the cut‐off values of sarcopenia‐related indices, including PhA (e.g., 4.1° for men and 3.6° for women in Japanese older adults [19]), were not employed in this study; we performed analyses given the evidence that exacerbation of sarcopenia‐related indices is associated with an increased risk of sarcopenia.

### 2.4 Cognitive Assessment Tool and Definition of MCI

Cognitive function was assessed using the Japanese version of the Montreal Cognitive Assessment (MoCA‐J). The MoCA‐J is a 30‐point scale and can assess the following six domains of cognitive functions: attention (six points): digit span forward and backward, letter A tapping, and serial‐7 subtraction; executive function (four points): letter fluency, trail making, and verbal abstraction; memory (five points): delayed recall; languages (five points): naming and sentence repetition; visuospatial abilities (four points): cube copy and clock drawing; and orientation (six points): orientation of date and place [20]. A domain of attention (digits forward and backward and a serial subtraction task) is involved in attention, concentration and working memory [21]. Episodic memory performance is well‐assessed by a domain of memory (a delayed recall task) [22]. An executive function domain (a letter fluency task) is related to semantic memory [22]. One point is added if the participants have less than 12 years of education. The MoCA‐J has good internal consistency and reliability, and the optimal cut‐off point of the MoCA‐J for screening MCI was 25/26 [21]; therefore, MCI was defined as a MoCA‐J score of less than 26 points in this study.

### 2.5 Statistical Analysis

All the statistical analyses were performed using SPSS version 28 (IBM Corp., Armonk, NY, USA). Data are presented as mean ± standard deviation (SD) or median (interquartile range) for continuous variables and as number (frequency percentage) for categorical variables. In all cases, a statistically significant probability (*p*) value of <0.05 was considered statistically significant.

Comparisons between the sexes were performed using a two‐sample *t* test or Mann–Whitney *U* test for continuous variables and chi‐squared tests for categorical variables. We then conducted multivariate logistic regression analyses to investigate the association between MCI and sarcopenia‐related indices for all participants and by sex. Odds ratios (ORs) and 95% confidence intervals (CIs) were calculated. Three models were created to analyse participants' data: unadjusted, sex‐adjusted and sex‐ and age‐adjusted. For analyses by sex, two models were created: unadjusted and age‐adjusted. We conducted a multivariate linear regression analysis to investigate the relationship between the MoCA‐J and sarcopenia‐related indices. The analysis was conducted using models similar to those of the multiple logistic regression analyses. Next, the correlation coefficients between the MoCA‐J domains and sarcopenia‐related indices were determined using Spearman's rank correlation analysis.

### 2.6 Sample Size

Since this was a cross‐sectional study using a database of a prospective cohort study (UMIN Study ID: UMIN000048056), the required sample size was not calculated in advance in this study. Instead, a total of 263 participants were finally included as the sample size for this study. Given the above study design, we addressed the statistical power by assessing the sample size using a post hoc power analysis.

## 3 Results

### 3.1 Characteristics of the Study Participants

**FIGURE 1:**
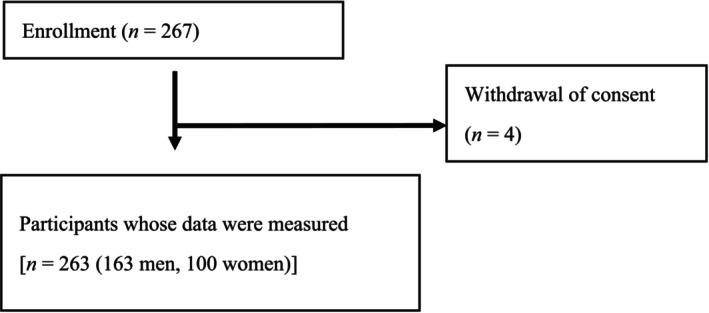
Study flow chart of participants.

Initially, 267 individuals were enrolled (Figure 1). Four participants withdrew consent prior to data collection. None of the participants had missing or incomplete data. Finally, 263 participants (163 men and 100 women) were included in the study.

Table 1 summarizes the characteristics of the participants. Participants aged ≥65 years included 58 men (35.6%) and 30 women (30.0%). SMI, ASM/BMI, HGS, HGS/USM and PhA were significantly lower in women than in men (all *p* < 0.001). Additionally, 82 participants (31.2%) had MCI (56 men [34.4%] and 26 women [26.0%]).

**TABLE 1:** Characteristics of the participants.

Variables	All (*n* = 263)	Men (*n* = 163)	Women (*n* = 100)	*p*
Age, year	59 [51–69]	60 [53–70]	58 [49–68]	0.225
≤12 years of education, *n* (%)	87 (33.1)	50 (30.7)	37 (37.0)	0.345
BMI, kg/m^2^	22.7 [21.0–24.9]	23.8 [22.0–25.4]	21.4 [19.4–23.3]	<0.001
SMI, kg/m^2^	7.5 ± 1.0	8.1 ± 0.8	6.5 ± 0.4	<0.001
ASM/BMI	0.92 [0.77–1.01]	0.98 [0.91–1.07]	0.76 [0.69–0.83]	<0.001
HGS, kg	32.2 ± 9.6	38.1 ± 7.0	22.7 ± 3.7	<0.001
HGS/USM, kg/kg	7.50 ± 1.11	7.77 ± 1.07	7.07 ± 1.03	<0.001
PhA, deg	5.2 ± 0.8	5.5 ± 0.7	4.7 ± 0.6	<0.001
Hypertension, *n* (%)	111 (42.2)	84 (51.5)	27 (27.0)	<0.001
Diabetes, *n* (%)	17 (6.5)	16 (9.8)	1 (1.0)	0.004
Dyslipidemia, *n* (%)	160 (60.8)	102 (62.6)	58 (58.0)	0.516
Current Smoker, *n* (%)	21 (8.0)	17 (10.5)	4 (4.0)	0.065
MoCA‐J, points	27 [25–28]	27 [25–28]	27 [25–28]	0.015
MCI, *n* (%)	82 (31.2)	56 (34.4)	26 (26.0)	0.172

*Note:* Data are presented as mean ± standard deviation, median [interquartile range], or number (frequency percentage).

Abbreviations: ASM, appendicular skeletal muscle mass; BMI, body mass index; HGS, handgrip strength; MCI, mild cognitive impairment; MoCA‐J, Japanese version of Montreal Cognitive Assessment; PhA, phase angle; SMI, skeletal muscle mass index; USM, upper extremity skeletal muscle mass.

### 3.2 Sarcopenia‐Related Indices Associated With MCI

**FIGURE 2:**
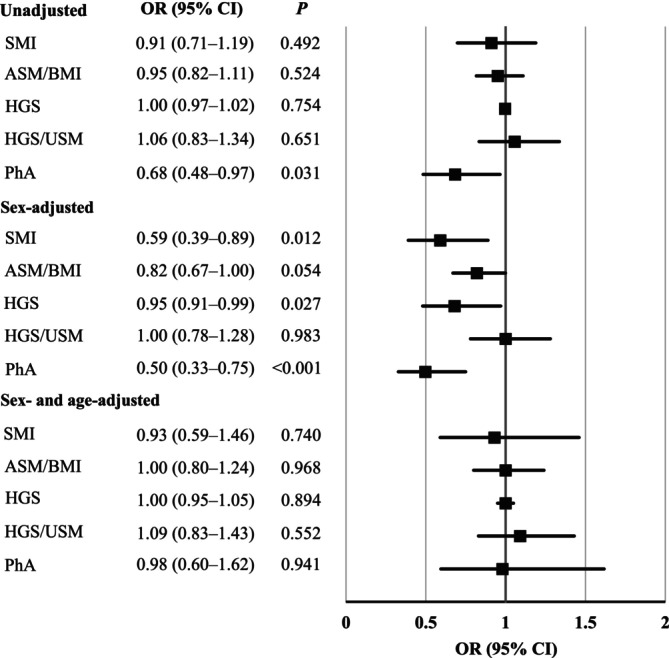
Association between a sarcopenia‐related index and MCI in all participants. Relationships between sarcopenia‐related indices and MCI are shown in unadjusted, sex‐adjusted and sex‐ and age‐adjusted models. MCI, mild cognitive impairment; OR, odds ratio; CI, confidence interval; SMI, skeletal muscle mass index; ASM, appendicular skeletal muscle mass; BMI, body mass index; HGS, handgrip strength; USM, upper extremity skeletal muscle mass; PhA, phase angle.

Figure 2 shows the associations between the sarcopenia‐related indices and MCI using logistic regression analysis for all participants. Only the PhA was significantly negatively associated with MCI in the unadjusted model (OR = 0.68, *p* = 0.031). In the sex‐adjusted model, SMI (OR = 0.59, *p* = 0.012), HGS (OR = 0.95, *p* = 0.027) and PhA (OR = 0.50, *p* < 0.001) were significantly negatively associated with MCI. However, in the sex‐ and age‐adjusted models, none of the five sarcopenia‐related indices were significantly associated with MCI.

**FIGURE 3:**
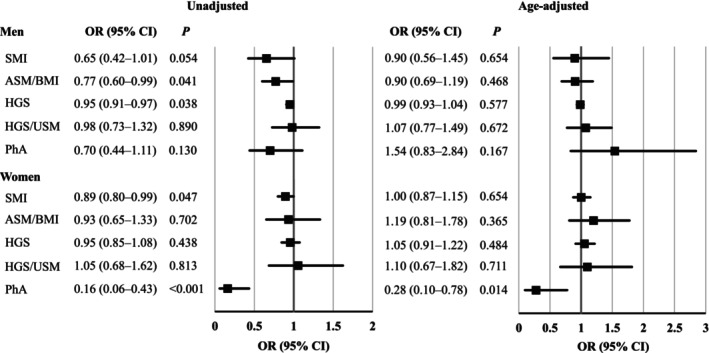
Association between a sarcopenia‐related index and MCI in subgroups categorized by sex. Relationships between sarcopenia‐related indices and MCI in the sex‐categorized subgroups are shown in unadjusted (left panel) and age‐adjusted (right panel) models. MCI, mild cognitive impairment; OR, odds ratio; CI, confidence interval; SMI, skeletal muscle mass index; ASM, appendicular skeletal muscle mass; BMI, body mass index; HGS, handgrip strength; USM, upper extremity skeletal muscle mass; PhA, phase angle.

Next, we investigated the associations between sarcopenia‐related indices and MCI using logistic regression analysis in the sex‐categorized subgroups (Figure 3). Among men, ASM/BMI (OR = 0.77, *p* = 0.041) and HGS (OR = 0.95, *p* = 0.038) were significantly negatively associated with MCI in the unadjusted model. However, these relationships were not statistically significant after adjusting for age. In women, SMI (OR = 0.89, *p* = 0.047) and PhA (OR = 0.16, *p* < 0.001) were significantly negatively associated with MCI in the unadjusted model. Only the PhA showed a significant negative association with MCI, even after adjusting for age (OR = 0.28, *p* = 0.014).

### 3.3 The Association Between PhA and Global Cognitive Function

Based on these results, we further investigated whether PhA was associated with cognitive function. Table 2 shows the associations between the PhA and MoCA‐J scores using multivariate linear regression analysis for all participants and subgroups categorized by sex. For all participants, PhA was significantly and positively associated with the MoCA‐J in the unadjusted (*β* = 0.15, *p* = 0.015) and sex‐adjusted models (*β* = 0.30, *p* < 0.001). However, these associations were not statistically significant after adjusting for age and sex. Similarly, PhA was not significantly associated with the MoCA‐J scores in men after adjusting for age, although a significant positive association was observed in the unadjusted model (*β* = 0.19, *p* = 0.015). Contrastingly, for women, PhA was significantly positively associated with MoCA‐J in both the unadjusted (*β* = 0.40, *p* < 0.001) and age‐adjusted models (*β* = 0.27, *p* = 0.005).

**TABLE 2:** Relationship between MoCA‐J and phase angle in all participants and in subgroups categorized by sex.

Model	All			Men			Women		
	*β*	95% CI	*p*	*β*	95% CI	*p*	*β*	95% CI	*p*
Unadjusted	0.15	0.03–0.27	0.015	0.19	0.04–0.34	0.015	0.40	0.21–0.57	<0.001
Sex‐adjusted	0.30	0.16–0.44	<0.001	—	—	—	—	—	—
Age‐adjusted	−0.03	−0.15–0.09	0.595	−0.09	−0.26–0.09	0.317	0.27	0.08–0.44	0.005
Sex‐ and age‐adjusted	0.06	−0.09–0.21	0.451	—	—	—	—	—	—

Abbreviations: CI, confidence interval; MoCA‐J, Japanese version of Montreal Cognitive Assessment; *β*, standardized coefficient.

### 3.4 Cognitive Subdomains Correlated With PhA

To further elucidate the relationship between PhA and cognitive function, we examined the correlation coefficients between PhA and the MoCA‐J subdomains (Table 3). PhA was significantly and positively correlated with memory in men (*r* = 0.24, *p* = 0.002). Furthermore, PhA was significantly and positively correlated with memory (*r* = 0.22, *p* = 0.031), language (*r* = 0.24, *p* = 0.015), executive function (*r* = 0.37, *p* < 0.001) and attention (*r* = 0.33, *p* < 0.001) in women.

**TABLE 3:** Relationship between MoCA‐J subdomains and phase angle in subgroups categorized by sex.

Subdomains of MoCA‐J	Men			Women		
	*r*	95% CI	*p*	*r*	95% CI	*p*
Memory	0.24	0.09–0.38	0.002	0.22	0.02–0.40	0.031
Language	0.03	−0.13–0.18	0.729	0.24	0.05–0.42	0.015
Executive function	0.10	−0.06–0.26	0.197	0.37	0.18–0.53	<0.001
Attention	0.06	−0.10–0.22	0.532	0.33	0.14–0.50	<0.001
Visuospatial abilities	0.05	−0.11–0.21	0.493	0.06	−0.14–0.26	0.542
Orientation	0.06	−0.10–0.21	0.480	0.01	−0.19–0.21	0.904

Abbreviations: CI, confidence interval; MoCA‐J, Japanese version of the Montreal Cognitive Assessment; *r*, correlation coefficient.

### 3.5 Statistical Power Analysis

In the logistic regression analysis in which MCI was the dependent variable, statistical significance was detected if OR was ≤0.5 or ≥2.0 when the relevant index (continuous variable) changed to the extent of one SD. The power was 99.8% for all participants (*n* = 263), 97.0% for men (*n* = 163) and 84.0% for women (*n* = 100).

In linear regression and correlation analyses, statistical significance was detected if a correlation coefficient was ≥0.25. The power was 98.7% for all participants (*n* = 263), 90.6% for men (*n* = 163) and 72.5% for women (*n* = 100).

## 4 Discussion

This is the first study to demonstrate that a higher PhA is associated with a lower risk of cognitive impairment in women in the general population. Furthermore, the increase in PhA was positively correlated with global cognitive function and cognitive subdomains, as assessed using the MoCA‐J. These findings suggest that improvements in muscle quality could contribute to a reduction in the risk of cognitive impairment, further highlighting the novel clinical significance of measuring PhA in preventing cognitive decline in women in the general population.

It has been reported that sarcopenia‐related indices are implicated in the development and progression of dementia [2, 8, 9, 10]. Changes in the levels of sex hormones with age, such as a decrease in oestrogen, especially in women, are also associated with the risk of dementia [23]. Accordingly, our results corroborate the possibility that the effects of ageing on muscle and sex hormones are more closely related to the increased risk of cognitive impairment in women compared to that in men. Moreover, PhA, which is associated with cognitive function in women in the general population, was the only sarcopenia‐related index examined in this study. This suggests that muscle quality plays a more significant role in cognitive function than other muscle properties/functions in women, although muscle mass and strength have been implicated in the risk of dementia [2, 10]. In this respect, our study revealed sex differences in the relationship between muscle properties/functions and cognitive function. Whereas muscle mass and strength, as well as quality, were lower in women than in men, only muscle quality was negatively associated with cognitive impairment in women; therefore, muscle quality would become more important in preventing cognitive impairment in women than in men. Conversely, it is possible that muscle mass, strength and quality compensate for each other to prevent cognitive impairment in men because there was no significant relationship between specific muscle properties/functions and cognitive function in men. Further studies are warranted to elucidate the mechanistic details underlying these sex differences, as well as why PhA is superior to other sarcopenia‐related indices as a potential marker for cognitive impairment. Nevertheless, our findings highlight the novel clinical significance of the PhA in identifying women with an increased risk of cognitive impairment in the general population.

In a recent report, we demonstrated that PhA is an indicator of muscle quality in young individuals and older adults [4]. Previous studies reported that PhA decreased with cognitive decline in patients with MCI and AD [11, 12]. It has also been suggested that the loss of intracellular fluids deleteriously affects cellular integrity and reduces PhA, leading to an unfavourable environment for muscle protein synthesis [24, 25]. Accordingly, it is possible that PhA is related to the ability to produce myokines that are implicated in cognitive function. In this respect, a recent study reported a significant relationship, in female but not in male patients with ad, between ad pathologies and levels of irisin [26], a myokine released from skeletal muscle during exercise that is involved in enhancing memory and learning [27]. The present study demonstrated similar sex differences in the relationship between PhA and cognitive function in the general population. A higher PhA was further positively correlated with memory in men in the general population and with several cognitive subdomains, including memory, in women. Therefore, PhA may reflect the ability to produce irisin and may be associated with cognitive function.

Memory decline is reportedly the earliest manifestation of cognitive impairment [28]. However, distinguishing abnormal forgetting from normal forgetting is challenging because forgetting is part of a normal experience and increases with age [28]. In this context, we found a positive correlation between memory and PhA in both men and women in the general population, whereas other cognitive subdomains were not significantly correlated with PhA in men. These findings suggest that PhA may be a potential surrogate marker of memory decline, thereby being useful in screening for pathological memory impairment, which is the earliest symptom of cognitive impairment in the general population.

PhA was positively correlated with other cognitive subdomains (language, executive function and attention) as well as memory in women in this study. This also supports the possibility that cognitive function in women is more affected by ageing‐related changes in sex hormones and muscle quality than that in men. Moreover, a recent study demonstrated that the volumes of the hippocampus, frontal lobe and cingulate were significantly lower in older adults with sarcopenia than in those without sarcopenia [29]. The hippocampus is involved in memory [30], and the frontal lobe plays a role in language [31] and executive function [30]. The prefrontal areas and cingulate are related to attention [30]. Accordingly, sarcopenia has detrimental effects on brain function [29], and PhA might reflect sarcopenia‐related early changes in brain structure.

Although molecular mechanisms underlying the relationship between sarcopenia and cognitive decline are yet to be elucidated, accumulating evidence focuses on the potential roles of the muscle–brain axis, which is the myokine‐related crosstalk between muscle and brain [32]. This further highlights the significance of PhA in myokine production because PhA is an indicator of cellular health [6]. These characteristics of PhA, which are involved in the muscle–brain axis, might confer higher sensitivity to cognitive decline on PhA compared to other sarcopenia‐related indices. The muscle–brain axis may be more prominent in women than in men, given the finding that the association between PhA and cognitive function was significant in women.

Since malnutrition and low physical performance are implicated in sarcopenia and dementia [11, 33], it is important to assess body composition. Conversely, individuals need to understand and follow the instructions when sarcopenia‐related indices are examined, such as grip strength and gait speed [12]. This raises concerns that sarcopenia‐related indices might not be measured appropriately in patients with dementia because cognitive decline might affect these tests deleteriously [12]; however, PhA obtained using BIA, an index of muscle quality [4], was found to be associated with cognitive function in patients with cognitive impairment [12]. Since there is no need for individuals subjected to BIA to follow the complex instructions, BIA would be appropriate for patients with cognitive impairment to assess physical decline by analysing PhA [12]. Moreover, a higher PhA assessed with BIA was associated with a decreased risk of cognitive impairment in women and memory impairment in both sexes in the general population in this study. In light of the noninvasive, reproducible and cost‐effective method of BIA using a portable device [11, 12, 33], these findings suggest that PhA measured using BIA is an implementable component of annual health checkups, which could contribute to identifying men and women with an increased risk of sarcopenia in the general population and women with a risk of cognitive decline as well as sarcopenia.

This study had some limitations. First, this study was not a longitudinal but a cross‐sectional study. Therefore, the observed relationships cannot be determined as causal. Second, stratified analyses based on certain factors, such as age, were not performed because of the limited sample size. Nevertheless, the sample size was sufficient in the logistic regression analysis and acceptable in linear regression and correlation analyses. Third, we did not measure the levels of myokines and sex hormones, which could provide further insight into the current understanding of this study. Fourth, the participants in this study were members of the general Japanese population aged ≥40 years, which might have caused unconscious bias. Finally, the findings of this study were obtained from analyses of data from the Japanese general population, which raises the concern that the appropriate selection of diagnostic criteria for sarcopenia would depend on race [1]. Nevertheless, we used the MoCA‐J as a cognitive test whose reliability and validity have been established [21]. Therefore, our findings would be helpful for other ethnicities, although caution is required in the extrapolation. Future longitudinal studies with larger sample sizes are warranted to address these issues and corroborate the findings of this study.

In conclusion, to our knowledge, this is the first study to demonstrate that PhA is a potential novel marker of cognitive decline in women in the general population. Measuring PhA could allow the identification of women with the risk of cognitive decline and sarcopenia, thereby highlighting the need to reduce the risk through early interventions for these individuals. PhA may also detect memory impairment in men at risk of cognitive decline. These findings may help develop novel strategies for preventing sarcopenia and cognitive impairment in the general population.

## Conflicts of Interest

The authors declare no conflicts of interest.
